# The relationship between salivary C-reactive protein and cognitive function in children aged 11–14 years: Does psychopathology have a moderating effect?

**DOI:** 10.1016/j.bbi.2017.07.002

**Published:** 2017-11

**Authors:** Alexis E. Cullen, Ben M. Tappin, Patricia A. Zunszain, Hannah Dickson, Ruth E. Roberts, Naghmeh Nikkheslat, Mizan Khondoker, Carmine M. Pariante, Helen L. Fisher, Kristin R. Laurens

**Affiliations:** a Department of Psychosis Studies, King’s College London, London, UK; b Section of Stress, Psychiatry and Immunology & Perinatal Psychiatry, Department of Psychological Medicine, King’s College London, London, UK; c Department of Forensic and Neurodevelopmental Sciences, King’s College London, London, UK; d MRC Social, Genetic & Developmental Psychiatry Centre, Institute of Psychiatry, Psychology & Neuroscience, King’s College London, London, UK; e ARC Centre of Excellence in Cognition and its Disorders, Department of Psychology, Royal Holloway, University of London, UK; f Norwich Medical School, University of East Anglia, UK; g School of Psychology, Australian Catholic University, Brisbane, Australia; h Research Unit for Schizophrenia Epidemiology, School of Psychiatry, University of New South Wales, Sydney, Australia; i Neuroscience Research Australia, Sydney, Australia

**Keywords:** Inflammation, Diurnal sampling, Neurocognitive function, Paediatric, Psychiatric symptoms, Psychosis, Depression

## Abstract

Elevated C-reactive protein (CRP), a non-specific biomarker of systemic bodily inflammation, has been associated with more pronounced cognitive impairments in adults with psychiatric disorders, particularly in the domains of memory and executive function. Whether this association is present in early life (i.e., the time at which the cognitive impairments that characterise these disorders become evident), and is specific to those with emerging psychiatric disorders, has yet to be investigated. To this end, we examined the association between salivary CRP and cognitive function in children aged 11–14 years and explored the moderating effect of psychopathology. The study utilised data from an established longitudinal investigation of children recruited from the community (N = 107) that had purposively over-sampled individuals experiencing psychopathology (determined using questionnaires). CRP was measured in saliva samples and participants completed assessments of cognition (memory and executive function) and psychopathology (internalising and externalising symptoms and psychotic-like experiences). Linear regression models indicated that higher salivary CRP was associated with poorer letter fluency (*β* = −0.24, *p* = 0.006) and scores on the inhibition (*β* = −0.28, *p* = 0.004) and inhibition/switching (*β* = −0.36, *p* < 0.001) subtests of the colour-word interference test, but not with performance on any of the memory tasks (working, visual, and verbal memory tasks). Results were largely unchanged after adjustment for psychopathology and no significant interactions between CRP and psychopathology were observed on any cognitive measure. Our findings provide preliminary evidence that elevated salivary CRP is associated with poorer cognitive function in early life, but that this association is not moderated by concurrent psychopathology. These findings have implications for early intervention strategies that attempt to ameliorate cognitive deficits associated with emerging psychiatric disorders. Further research is needed to determine whether salivary CRP levels can be used as a valid marker of peripheral inflammation among healthy adolescents.

## Highlights

• Salivary CRP predicts poorer executive functioning in children aged 11–14 years.
• The association is not confounded or moderated by concurrent psychopathology.
• Findings have implications for interventions targeting cognitive deficits.
• Salivary CRP can be used to investigate inflammation-brain function relationships.

## 1 Introduction

Over the past decade, there have been increased efforts to elucidate the role of inflammatory processes in both the pathogenesis and symptomology of psychiatric disorders (Haroon et al., 2012). These efforts have demonstrated that a number of psychiatric disorders, including schizophrenia, bipolar disorder, and depression, are characterised by elevated concentrations of C-reactive protein (CRP), a non-specific biomarker of systemic bodily inflammation (Fernandes et al., 2016a, Fernandes et al., 2016b, Howren et al., 2009). Moreover, within these populations, elevated CRP is associated with poorer cognitive function. Specifically, higher CRP has been found to correlate with greater cognitive impairments, particularly in memory and executive function domains, among individuals with schizophrenia (Bulzacka et al., 2016, Dickerson et al., 2007, Frydecka et al., 2015, Johnsen et al., 2016, Micoulaud-Franchi et al., 2015); with poorer memory and general cognitive function in those with bipolar disorder (Dickerson et al., 2013); and with attention and executive function deficits among individuals with depression (Chang et al., 2012, Krogh et al., 2014). Such findings lend support to the notion that chronic inflammation may contribute to the cognitive impairments that typically characterise these disorders; however, two major questions remain unanswered: Firstly, to what extent is the association between CRP and cognition specific to those with psychiatric disorders? Secondly, is this association present during early life? The latter is particularly pertinent given that the cognitive impairments observed among individuals with these disorders emerge during childhood, many years before disorder onset (Dickson et al., 2012, Koenen et al., 2009, Laurens et al., 2015).

Studies examining the relationship between CRP and cognition among those without psychiatric disorders have typically focused on distinct sub-populations characterised by cognitive impairments. Much research has focused on healthy older adults (≥50 years); both cross-sectional and longitudinal studies indicate that elevated CRP is associated with poorer cognitive function in this population (Jenny et al., 2012, Marioni et al., 2009, Noble et al., 2010, Schram et al., 2007, Teunissen et al., 2003), though the association is rendered non-significant after adjustment for sociodemographic factors in some studies (Alley et al., 2008, Dik et al., 2005, Kao et al., 2011). Whilst a small number of these studies assessed depression symptoms, none examined the effect of these symptoms on the relationship between CRP and cognition; thus, the extent to which this relationship might be explained by psychiatric morbidity is unknown. Other studies have focused on populations with specific medical conditions. In cross-sectional investigations, CRP has been associated with poorer cognitive ability (again, particularly in the domains of memory and executive function) in preterm infants (O'Shea et al., 2013, Rose et al., 2016), infants with congenital heart problems (Li et al., 2014), children with obstructive sleep apnoea (Huang et al., 2016), and in adults experiencing mild traumatic brain injury (Su et al., 2014) or chronic obstructive pulmonary disease (Crisan et al., 2014). Inconsistent findings characterise studies examining populations that are neither exclusively older in age nor characterised by physical health conditions. A study of adults aged 18–82 years reported that CRP was not associated with executive function abilities (Schuur et al., 2010), whilst another large study of 28–91 year olds observed negative correlations between CRP and slower processing speed (but not executive function or memory performance) among African American (but not European American) participants (Windham et al., 2014). Only two studies, both longitudinal, have focused on young individuals from the general population; CRP did not predict memory or executive function in a two-year follow-up of adolescents (Jonker et al., 2014) or global cognition in a 13-year follow-up of young adults (Cohen-Manheim et al., 2015).

Thus, whilst elevated CRP has been associated with greater cognitive impairment in some non-psychiatric samples, the association appears to be restricted to populations in which cognitive impairment is also prevalent (e.g., older adults and those with physical health conditions). In contrast, a lack of evidence exists to suggest that CRP predicts later cognitive function in adolescents and young adults in the general population, which may reflect follow-up periods exceeding two years that were employed in these studies. However, the only previous study of adolescents did not investigate whether the relationship was present among those experiencing psychopathology (e.g., depression or subclinical psychotic symptoms). This is important, as opportunities for intervening to improve cognitive deficits associated with psychiatric disorders (either by psychological or biological means) are likely to be maximised in early life.

In light of these issues, the current study sought to investigate associations between inflammation, cognitive impairments, and emerging psychopathology in childhood, utilising salivary CRP as a marker of inflammation. Salivary CRP offers a less-invasive alternative to blood-based CRP measurement, particularly in child/adolescent populations in which there is often reluctance to provide blood samples. With the exception of a single study of healthy adults that failed to find an association between salivary and serum CRP (Dillon et al., 2010), most previous studies have demonstrated moderate-to-high correspondence between CRP levels in serum/plasma and saliva (Byrne et al., 2013, Iyengar et al., 2014, Ouellet-Morin et al., 2011, Out et al., 2012). The linear relationship observed between CRP in blood and saliva in these studies implies that the latter is a valid marker of peripheral inflammation. Nonetheless, the origin of salivary CRP is currently unclear. It has been proposed that CRP derived from saliva samples may reflect systemic CRP (predominately produced by the liver) that has leaked into gingival crevicular fluid via gingival tissues, though salivary CRP might also be produced by gingival tissues in response to periodontal infections (Salimetrics, 2011). By utilising salivary CRP as a marker of inflammation, the current study provides an opportunity to determine whether associations observed between blood-derived CRP and cognition extend to salivary CRP.

Capitalizing on a well-established longitudinal study of children recruited from the community (Laurens and Cullen, 2016), we examined the effect of salivary CRP on measures of memory and executive function in a sample of children aged 11–14 years that was enriched for children presenting with psychopathology (as determined using screening questionnaires). We first examined the predictive effect of salivary CRP on memory and executive function after adjusting for various methodological and participant factors that might confound the association. We then explored interactions between salivary CRP and psychopathology domains (internalising and externalising symptoms and psychotic-like experiences) to determine whether any associations between CRP and cognitive performance differed among children with and without psychopathology. We predicted that, in this sample of adolescents enriched for psychopathology, higher salivary CRP would be associated with poorer memory and executive function. We also anticipated that concurrent psychopathology would moderate the association between CRP and these cognitive functions.

## 2 Materials and methods

### 2.1 Participants and procedure

The current study uses data from an established longitudinal investigation of children initially recruited from the general population of South London at age 9–12 years (Laurens and Cullen, 2016, Laurens et al., 2007, Laurens et al., 2011). From the community sample of 1343 children and caregivers completing screening, a subset of these participants were selected (N = 123) to participate in laboratory-based assessments conducted at the Institute of Psychiatry, Psychology & Neuroscience, King’s College London, UK. This intensely-studied sample was enriched with children presenting with psychopathology [developmental delays (Laurens et al., 2007); social, emotional, and/or behavioural problems (Goodman, 2001); and psychotic-like experiences (Laurens et al., 2012)] and/or a family history of psychosis [confirmed using the Family Interview for Genetic Studies (Maxwell, 1992)]; the sample also included unaffected children without psychopathology or family history of psychosis (44% of those initially recruited to the study). Children with a neurological condition (e.g., epilepsy or cerebral palsy), autism, Asperger’s disorder, or a diagnosed learning disability were not eligible to participate. Laboratory assessments comprising a variety of biological and psychosocial measures were completed biennially throughout adolescence in this cohort. The current study examines data obtained at a follow-up assessment completed at age 11–14 years, and includes participants who provided at least one saliva sample at this assessment phase and completed the cognitive measures described below (*N* = 107).

At this assessment phase, children and their caregivers provided written informed assent and consent, respectively, for participation. Ethical permission for the study was granted by the Joint South London and Maudsley and the Institute of Psychiatry NHS Research Ethics Committee. Caregivers provided information on family psychiatric history and participant ethnicity [determined using information obtained using the Family Interview for Genetic Studies (Maxwell, 1992)] and caregiver occupation [coded according to the UK National Statistics Socio-economic Classification (Office for National Statistics, 2010)]. Children completed the Pubertal Developmental Scale questionnaire (Carskadon and Acebo, 1993) and provided information on tobacco and cannabis use via a self-report measure (McVie and Bradshaw, 2005). Height and weight were collected by the researcher at the assessment session to compute Body Mass Index (BMI; kilograms/metres^2^). The LMS method (Pan and Cole, 2012) was used to convert raw BMI data into age- and sex-adjusted standard deviation scores (z scores) using UK growth reference data (Cole et al., 1998). Participant BMI z scores were then classified according to clinical centiles as follows: very underweight (≤0.4th), low weight (≤2nd), healthy weight (>2 to <91st), overweight (≥91st), obese (≥98th), and extremely obese (≥99.6th). Obese and extremely obese categories were combined for the purposes of the current study.

### 2.2 Salivary C-reactive protein

#### 2.2.1 Sample collection procedure

As described previously (Cullen et al., 2014), participants received written instructions for collecting saliva samples at home using the passive drool procedure recommended for CRP measurement (http://www.salimetrics.com). Participants were asked to collect six saliva samples throughout the day on two consecutive days: at awakening, and at 15, 30, and 60 min after awakening, and at 12:00 h, and 20:00 h. Participants were instructed to wake before 10:00 h and collect the first sample immediately upon awakening, to avoid food consumption for 30 min prior to each sample collection, and to refrain from strenuous exercise. Using a sampling diary, participants recorded the time of each sample collection, time of awakening, and described their activities during the day. Participants were also asked to indicate whether they had experienced any general health problems (i.e., headache, tiredness, cold, infection, hay fever, allergies, asthma) or dental problems (i.e., toothache, mouth ulcer, bleeding gums) during the past week, including the days of sample collection, via a self-report checklist. Samples were stored in the participant’s home freezer until collection, and subsequently frozen at −20 °C at the laboratory for up to five years prior to assay (mean lapse of time = 1186 days).

#### 2.2.2 Salivary CRP assay

Salivary CRP levels were measured using a commercially available enzyme-linked immunoassay kit from Salimetrics, Suffolk, UK, following the recommended procedure. Frozen saliva samples were allowed to thaw at room temperature and centrifuged at 3000 rpm for 15 min to remove mucins. Samples were diluted in the manufacturer’s buffer (1:10) and assayed in duplicate, using the Tecan Freedom Evo 100 automated liquid handler. After 2 h of incubation on a microplate coated with anti-CRP antibodies, in the presence of diluted enzyme conjugate solution linked to horseradish peroxidase, wells were washed four times. A solution of tetramethylbenzidine was then added and the plate was incubated in the dark for 30 min with continual mixing. After adding a stop solution of sulphuric acid and further mixing on a plate rotator for 3 min, optical density was read on a Beckman Coulter DTX 880 plate reader, with Multimode Detection Software 2.0.012, at 450 nm with correction at 620 nm. SoftMax Pro 4.8 software was used to calculate the CRP values, following a 4-parameter fit. The coefficient of variation (CV, calculated as standard deviation * 100/mean) was calculated for each sample to determine the degree of variation between duplicates.

#### 2.2.3 CRP sample availability and effect of methodological factors

Participants were instructed to collect 12 samples over a two-day period, but participant non-compliance (i.e., failure to collect all samples) or insufficient saliva available for assay after aliquoting reduced the mean number of saliva samples available for CRP assay to 5.67 per participant (N = 659; range: 1–12). Rather than deriving an average CRP value across all time-points (which might be influenced by the fact that participants were missing data for different time-points), we used the samples obtained at 12:00 on Day 1 to examine the association between salivary CRP and cognition as these were the most prevalent. To establish whether it was appropriate to use another sample in place of the 12:00 h Day 1 sample (where this sample was unavailable), we performed preliminary analyses on the total dataset (N = 605 after excluding 52 samples with CV values > 20% and 2 samples with extremely high CRP values) to determine whether CRP values were influenced by time and day of sampling. In this larger dataset, we also investigated the influence of participant demographic factors on CRP levels in order to identify potential confounders in the main analysis.

These preliminary methodological analyses are detailed in the Supplementary Material. In brief, a mixed effects linear regression model indicated a significant effect of sampling time on salivary CRP levels (CRP values for all other time-points significantly lower relative to the awakening sample, *p* ≤ 0.02) and a significant effect of day of sampling (CRP levels lower on the second day of collection, *p* < 0.001). Neither time of awakening nor length of time in storage were associated with CRP. With regards to participant factors, after adjustment for time and day of sampling, those who reported recent dental problems were characterised by higher CRP (*p* = 0.01); however, CRP was not significantly associated with age, BMI, sex, ethnicity, socioeconomic status, recent health problems, or a family history of psychosis. Thus, where a 12:00 h Day 1 sample was unavailable, data were replaced with any non-awakening sample obtained on Day 1 (*n* = 8), a 12:00 h sample collected on Day 2 (*n* = 11), or any other Day 2 sample except the awakening sample (*n* = 7). Blood CRP values were estimated using a regression equation (*y* = 1553.15 *x* − 1413.19; where *y* = serum CRP and *x* = salivary CRP) reported in a previous study examining the association between salivary and serum CRP in healthy adults (Ouellet-Morin et al., 2011). Estimated blood CRP values were calculated in order to identify high estimated CRP values [blood CRP > 10 mg/L (Pearson et al., 2003)] likely to be indicative of acute inflammation. All estimated blood CRP values were less than 10 mg/L.

### 2.3 Cognitive assessments

Participants completed a battery of cognitive assessments at the research session (typically saliva samples were collected by participants during the same week, mean lapse of time between cognitive assessment and saliva collection was ±1.4 months). Selected subtests from the Wide Range Assessment of Memory and Learning 2nd Edition [WRAML2 (Sheslow and Adams, 2003)] and the Delis–Kaplan Executive Function System [D-KEFS (Delis et al., 2001)] were used to assess memory and executive function. Table 1 provides a description of each WRAML2 and D-KEFS subtest administered. Standardised scores were derived for each subtest using manual-reported population norms.

**Table 1:** Memory and executive function measures completed by participants.

Cognitive measure and subtest	Normative data		Subtest description
	Mean	(SD)	
Memory: WRAML2 subtests			
Number-letter	10	(3)	Repeating strings of numbers and letters presented orally
Verbal memory index	100	(15)	
Story memory			Immediate recall of two short stories presented orally
Verbal learning			Immediate free recall of a list of words presented orally
Visual memory index	100	(15)	
Design memory			Drawing five visually-presented geometric designs
Picture memory			Identifying differences between four similar pairs of pictures
Verbal working memory	10	(3)	Immediate recall of word lists by category (animal vs. non-animal)
Executive function: D-KEFS subtests			
Verbal fluency test			
Letter fluency	10	(3)	Generating words beginning with F, A, and S within 60 s
Category fluency	10	(3)	Generating items from categories (animals and boys names) within 60 s
Colour-word interference test			
Inhibition	10	(3)	Naming the ink colour of colour-words presented in contrasting coloured ink
Inhibition/switching	10	(3)	Alternating between the Inhibition condition described above and naming the colour-word whilst ignoring ink colour
Towers test			
Total achievement score	10	(3)	Building towers using one to five disks in the fewest possible moves

Note. WRAML2: Wide Range Assessment of Memory and Learning 2nd edition; D-KEFS: Delis–Kaplan Executive Function System.

### 2.4 Psychopathology assessments

Children completed the Youth Self-Report (YSR) for school-aged children (Achenbach and Rescorla, 2001), a widely-used measure of psychopathology that has been evaluated extensively in child and adolescent populations and exhibits high reliability and validity. The YSR incorporates a 112-item checklist of problems occurring during the past six months, each item is scored on a three-point scale (0 ‘not true’, 1 ‘somewhat true or sometimes true’, or 2 ‘very true or often true’). Item scores can be summed to derive eight empirically-derived syndrome subscales: (i) anxious/depressed, (ii) withdrawn/depressed, (iii) somatic complaints, (iv) social problems, (v) thought problems, (vi) attention problems, (vii) rule-breaking behaviour, (viii) aggressive behaviour; subscale scores can then be combined to obtain two overall scores indexing internalising (subscales i, ii, and iii) and externalising problems (subscales vii and viii). The YSR provides age- and sex-adjusted normative data that can be used to categorise internalising and externalising scale scores as falling within the ‘normal’, ‘borderline’, or ‘clinical’ ranges. For the purposes of the current study, we combined the ‘borderline’ and ‘clinical’ groups to create binary variables (normal vs. borderline/clinical) for both the internalising and externalising subscales.

Participants additionally completed a nine-item psychotic-like experiences measure (Laurens et al., 2012, Laurens et al., 2007). This measure included a range of hallucination- and delusion-like experiences, each rated on a three-point scale (0 ‘not true’, 1 ‘somewhat true’, or 2 ‘certainly true’). A binary variable was derived indicating the presence of at least one experience rated somewhat or certainly true.

### 2.5 Statistical analyses

Analyses were conducted using Stata version 12 (StataCorp, 2011). Cognitive scores, but not salivary CRP scores, were approximately normally distributed in the current sample (N = 107). Applying a square root transformation to the salivary CRP variable improved the skewness but not the kurtosis (values 0.47 and 4.20, respectively), therefore all regression analyses using the transformed CRP variable were performed with robust standard errors. Linear regression analyses were conducted to examine the association between CRP (independent variable: IV) and each cognitive subtest examined (dependent variable: DV); as noted above, all regression analyses were adjusted for sampling day and recent dental problems owing to the association of these variables with salivary CRP (see Supplementary Material). Standardised beta values (*β*) are presented for ease of comparison across cognitive subtests. In supplementary analyses, the association between each psychopathology variable (IV) and CRP (DV) was examined (Supplementary Material). We then repeated all analyses examining the effect of CRP on cognition with current psychopathology included as additional covariates; in the final step, interactions between each psychopathology variable and CRP were included in each model.

## 3 Results

### 3.1 Sample characteristics

Demographic characteristics of the sample are displayed in Table 2. The mean age of participants at the time of CRP collection was 13.2 years and approximately half (48.1%) were male. Consistent with the ethnic diversity known to characterise the population from which participants were sampled, one third were of white British ethnicity. Just over one quarter (27.1%) had a first- or second-degree relative with a psychotic disorder (schizophrenia, schizoaffective disorder, or bipolar disorder). With regards to current psychopathology, 17.1% and 10.5% of participants scored in the borderline/clinical range on the YSR internalising and externalising scales, respectively, and 43.0% of the sample reported at least one somewhat/certainly true psychotic-like experience.

**Table 2:** Sample demographics.

	Total sample (*N* = 107)	
Age (years); mean (SD)	13.2	(1.1)
Male sex; *n* (%)	51	(48.1)
BMI (kilograms/metres^2^); mean (SD)	19.8	(3.2)
BMI categories		
Healthy weight	84	(80.8)
Overweight	15	(14.4)
Obese	5	(4.8)
Ethnicity; *n* (%)		
White British	35	(32.7)
White other	28	(26.2)
Black African or Caribbean	12	(11.2)
Other	32	(29.9)
Socioeconomic status based on parental occupation; *n* (%)		
Higher managerial, administrative, and professional	67	(62.6)
Intermediate	26	(24.3)
Routine and manual	14	(13.1)
Tobacco use; *n* (%)	2	(1.9)
Cannabis use; *n* (%)	3	(2.8)
Family history of psychosis; *n* (%)^b^	29	(27.1)
Salivary CRP (raw) pg/ml; mean (SD)	2027.6	(1070.2)
Salivary CRP (SQRT transformed) pg/ml; mean (SD)	43.6	(11.3)
YSR Internalising ‘borderline/clinical’ range; *n* (%) ^c^	18	(17.1)
YSR Externalising ‘borderline/clinical’ range; *n* (%) ^d^	11	(10.5)
Reported ≥ 1 ‘somewhat true’ psychotic-like experience, *n* (%) ^e^	46	(43.0)
WRAML2: Number letter; mean (SD)	12.2	(3.2)
WRAML2: Verbal memory; mean (SD)	106.7	(12.7)
WRAML2: Visual memory; mean (SD)	90.7	(13.2)
WRAML2: Verbal working memory; mean (SD)	10.2	(2.3)
D-KEFS: Letter fluency; mean (SD)	10.6	(2.7)
D-KEFS: Category fluency; mean (SD)	12.5	(3.1)
D-KEFS: CW Inhibition; mean (SD)	11.1	(2.3)
D-KEFS: CW Inhibition/switching; mean (SD)	10.8	(2.3)
D-KEFS: Towers test; mean (SD)	11.4	(1.9)

Note. SD, standard deviation; BMI: Body Mass Index; CRP: C-reactive protein; SQRT: square root; pg/ml: picograms per millilitre; YSR: Youth Self-Report; WRAML2: Wide Range Assessment of Memory and Learning 2nd edition; D-KEFS: Delis–Kaplan Executive Function System; CW: colour-word interference test. ^a^Includes schizophrenia and schizoaffective disorder. ^b^YSR Internalising: ‘borderline’ (n = 6); ‘clinical’ (n = 12); ^c^YSR Externalising: ‘borderline’ (n = 4); ‘clinical’ (n = 7). ^e^No. of somewhat or certainly-true PLEs reported: 1 (n = 18); 2 (n = 15); 3 (n = 4); 4 (n = 5); 5 (n = 2); 6 (n = 1); 8 (n = 1). Missing data: BMI (n = 3); WRAML2 number letter (n = 3) WRAML2 verbal and visual memory indices (n = 1); WRAML2 verbal working memory (n = 1); D-KEFS letter and category fluency (n = 2); D-KEFS CW inhibition and inhibition/switching (n = 3); D-KEFS tower test (n = 2); YSR internalising (n = 2); YSR externalising (n = 2).

### 3.2 Predictive effect of CRP on cognitive performance

Table 3 presents the results of linear regression analyses examining the association between salivary CRP and cognitive performance after adjustment for methodological and demographic factors (sampling day and recent dental problems). Analyses indicated a significant association between CRP and several measures of executive functioning; specifically, higher salivary CRP was associated with lower scores on the letter fluency subtest of the verbal fluency test (*β* = −0.24, *p* = 0.006), with a statistical trend observed also for lower category fluency subtest scores (*β* = −0.17, *p* = 0.08). Additionally, higher CRP levels were associated with significantly poorer performance on both the inhibition (*β* = −0.28, *p* = 0.004) and inhibition/switching (*β* = −0.36, *p* < 0.001) subtests of the colour-word interference test. In contrast, CRP was not significantly associated with scores on the D-KEFS towers test or performance on any of the WRAML2 memory measures.

**Table 3:** Associations between C-reactive protein (CRP) and cognitive performance in 11–14 year-olds.

	CRP adjusted for methodological/participant factors^a^				CRP adjusted for methodological/participant factors and psychopathology^b^				Interaction effects for psychopathology * CRP
	*β*	*B*	(95% CI)	*p*	*β*	*B*	(95% CI)	*p*	*β, p*
WRAML2: Number letter	−0.14	−0.04	(−0.09 to 0.01)	0.14	−0.15	−0.04	(−0.09 to 0.01)	0.09	INT*CRP (0.13, 0.73) EXT*CRP (0.03, 0.88) PLE*CRP (0.24, 0.54)
WRAML2: Verbal memory	0.01	0.01	(−0.19 to 0.20)	0.94	0.03	0.03	(−0.16 to 0.22)	0.77	INT*CRP (0.05, 0.88) EXT*CRP (0.09, 0.71) PLE*CRP (0.39, 0.26)
WRAML2: Visual memory	−0.05	−0.06	(−0.33 to 0.21)	0.68	−0.06	−0.07	(−0.33 to 0.19)	0.60	INT*CRP (0.25, 0.45) EXT*CRP (0.06, 0.88) PLE*CRP (0.30, 0.51)
WRAML2: Verbal working memory	−0.02	−0.00	(−0.05 to 0.05)	0.89	−0.01	−0.00	(−0.05 to 0.04)	0.90	INT*CRP (−0.41, 0.42) EXT*CRP (0.05, 0.92) PLE*CRP (−0.05, 0.93)
D-KEFS: Letter fluency	−**0.24**	−**0.06**	**(**−**0.10 to** −**0.02)**	**0.006**	−**0.23**	−**0.06**	**(**−**0.10 to** −**0.01)**	**0.009**	INT*CRP (−0.41, 0.22) EXT*CRP (0.02, 0.92) PLE*CRP (0.12, 0.74)
D-KEFS: Category fluency	−0.17	−0.05	(−0.10 to 0.01)	0.08	−0.17	−0.05	(−0.10 to 0.01)	0.10	INT*CRP (0.17, 0.78) EXT*CRP (−0.05, 0.92) PLE*CRP (0.38, 0.35)
D-KEFS: CW Inhibition	−**0.28**	−**0.05**	**(**−**0.09 to** −**0.02)**	**0.004**	−**0.25**	−**0.05**	**(**−**0.09 to** −**0.01)**	**0.01**	INT*CRP (0.03, 0.96) EXT*CRP (0.40, 0.32) PLE*CRP (0.06, 0.87)
D-KEFS: CW Inhibition/switching	−**0.36**	−**0.07**	**(**−**0.11 to** −**0.04)**	**<0.001**	−**0.35**	−**0.07**	**(**−**0.11 to** −**0.03)**	**<0.001**	INT*CRP (0.03, 0.92) EXT*CRP (0.55, 0.11) PLE*CRP (−0.16, 0.59)
D-KEFS: Towers test	−0.04	−0.01	(−0.05 to 0.04)	0.77	−0.04	−0.01	(−0.05 to 0.03)	0.74	INT*CRP (1.02, 0.13) EXT*CRP (-0.11, 0.82) PLE*CRP (-0.21, 0.69)

Note. WRAML2: Wide Range Assessment of Memory and Learning 2nd edition; D-KEFS: Delis–Kaplan Executive Function System; CW: colour-word interference test; *β* standardised beta coefficient from linear regression analysis; *B*: unstandardised regression coefficients; CI: confidence interval. INT: internalising symptoms; EXT: externalising symptoms: PLE: psychotic-like experiences. All analyses performed with robust standard errors using square root transformed CRP. ^a^Adjusted for day of saliva sample collection (Day 1 vs. Day 2) and recent dental problems; ^b^Additionally adjusted for current psychopathology (Youth Self-Report internalising scores in borderline or clinical range; Youth Self-Report externalising scores in borderline or clinical range; presence of at least one somewhat true psychotic-like experience).

Bold indicates statistical significance at the 0.05 level.

### 3.3 Association between current psychopathology and CRP

Linear regression analyses, adjusted for sample day and recent dental problems, indicated that internalising problems, externalising problems, and psychotic-like experiences were not significantly associated with salivary CRP (*p* > 0.05, Supplementary Material).

### 3.4 Effect of current psychopathology on the association between CRP and cognition

Analyses were next performed to examine the association between salivary CRP and cognitive scores after additionally adjusting for psychopathology variables. As shown in Table 3, the pattern of results was largely unchanged; salivary CRP remained significantly associated with scores on the D-KEFS letter fluency subtest of the verbal fluency test and the D-KEFS inhibition and inhibition-switching subtests of the colour-word interference test (*p* < 0.01 for all). The association between CRP level and D-KEFS category fluency subtest scores, however, reduced in significance although the standardised beta value did not change (*β* = −0.17, *p* = 0.10).

### 3.5 Moderating effects of psychopathology

As a final step, interaction terms between CRP and each of the three psychopathology domains (internalising and externalising symptoms and psychotic-like experiences) were additionally included in each model; however, as no significant interaction effects were observed (see Table 3), these were dropped from the final models.

### 3.6 Consideration of additional factors

All analyses were repeated after including lapse of time between cognitive assessment and salivary CRP sampling, with no change to the overall pattern of results (i.e., all previously reported statistically significant results remained significant). Only one participant in the sample may have recently used psychotropic medication (stimulant medication had been prescribed to this participant at the current follow-up assessment, which may have been used in the days preceding saliva collection); however, none of the participants had taken medication on either of the saliva collection days. CRP data for this participant were not outliers within the current sample and the results of the main analyses examining the relationship between salivary CRP and cognition were unchanged after excluding this participant.

## 4 Discussion

In this sample of children aged 11–14 years, enriched for those presenting with psychopathology, we examined the association between salivary CRP and cognitive performance, and, for the first time, the extent to which this relationship was modified by concurrent psychopathology. Consistent with previous studies examining CRP from blood samples, we showed that higher salivary CRP levels are associated with poorer performance on several executive functioning measures, thereby demonstrating the potential utility of salivary CRP in populations where there is reluctance to provide blood samples. However, salivary CRP was not associated with scores on memory subtests. The pattern of findings was unchanged after adjusting for current psychopathology (internalising and externalising symptoms and psychotic-like experiences) and we observed no significant interactions between salivary CRP and these psychopathology domains. Thus, the current findings provide no evidence to suggest that the association of salivary CRP and cognitive function is restricted to, or more pronounced among, adolescents characterised by emerging psychopathology.

Our findings should be interpreted cautiously owing to our use of salivary CRP alone as a measure of systemic inflammation. Studies examining the concordance between salivary CRP and blood-derived CRP values have yielded mixed findings; whilst most studies have shown moderate-to-high correlations (Byrne et al., 2013, Iyengar et al., 2014, Ouellet-Morin et al., 2011, Out et al., 2012) others have not (Dillon et al., 2010). It is important to note that only one of these studies examined adolescents (Byrne et al., 2013). As that study observed significant associations between salivary and serum CRP among adolescents with high serum CRP, but not those with low serum CRP, the extent to which salivary CRP is a valid marker of systemic inflammation in our sample of relatively healthy adolescents is unclear. In light of these findings, we do not advocate that researchers measure CRP in saliva only, but rather, when possible, measure both salivary and blood CRP levels in order to further test the extent to which salivary CRP represents a valid, non-invasive biomarker of systemic inflammation.

Our extended analyses indicated that salivary CRP levels were not significantly elevated among children classified as obese (see Supplementary Material), a finding that has been observed in previous studies of children aged 7–12 years (Goodson et al., 2014, Naidoo et al., 2012). In both previous studies, participant BMI categories were derived from US growth charts (Ogden et al., 2002) which differ from the British 1990 categories utilised in the current study (Lang et al., 2011), potentially contributing to our divergent finding. Alternatively, the fact that only a small proportion of our sample were classified as obese (4.8%) may have limited our ability to detect significant elevations in CRP in this group. Our use of salivary CRP also meant that we were unable to definitively identify participants with high CRP values, indicative of acute systemic infection or illness. Whilst we attempted to estimate blood CRP levels using previously-published data (Ouellet-Morin et al., 2011), further studies are needed to confirm the validity of this approach. Nonetheless, we observed robust associations between salivary CRP and cognitive function that mirrored those reported in studies utilising blood-derived CRP.

The current study is the first to demonstrate that CRP measured in saliva is predictive of poorer performance on several measures of executive function. Our findings obtained in this sample of children enriched for psychopathology are consistent with previous studies in which significant associations between blood-derived CRP levels and executive function have been observed among individuals with psychiatric disorders (Dickerson et al., 2013, Krogh et al., 2014, Micoulaud-Franchi et al., 2015), older adults (Marioni et al., 2009, Schram et al., 2007), and children with obstructive sleep apnoea (Huang et al., 2016). Contrary to our hypotheses, salivary CRP levels were unrelated to performance on any of the memory measures examined in the current study. This is surprising given the wealth of studies that have previously found CRP to be associated with memory performance in both psychiatric samples and older adults (Bulzacka et al., 2016, Dickerson et al., 2013, Frydecka et al., 2015, Johnsen et al., 2016, Marioni et al., 2009, Noble et al., 2010, Teunissen et al., 2003). It is possible that, among healthy adolescents, elevated CRP is specifically related to executive function but not memory performance. As discussed in detail below, the specific executive function domains examined in the current study might be served by brain regions which are particularly sensitive to the effects of elevated CRP during adolescence. Alternatively, it may be that our cognitive measures did not capture the specific impairments in memory that might be associated with elevations in CRP.

Our finding that psychopathology had neither a confounding nor moderating effect on the association of CRP and cognitive performance is in contrast to our hypotheses. Given that CRP has been associated consistently with cognition in samples of individuals with psychiatric disorders (schizophrenia, bipolar disorder, and depression), but not in samples of healthy individuals, we expected that the relationship between CRP and cognition would be more prominent among those with concurrent psychopathology. We found no evidence to support this. This may be due to the fact that the indices of psychopathology that we examined in the current study (i.e., scores in the borderline/clinical range of the YSR and the presence of at least one somewhat/certainly true psychotic-like experience) were of insufficient severity to moderate the effect of CRP on cognitive function. It is possible that clinically-determined psychiatric disorders may show interaction effects with CRP. Alternatively, CRP may be predictive of poor cognitive performance irrespective of concurrent psychopathology. Given that this appears to be the first study to examine potential interaction effects between CRP and psychopathology domains, it will be important to conduct similar analyses in larger samples to more definitively address these questions.

To our knowledge, only one previous study has examined the association of CRP and cognitive function in adolescents recruited from the general population (Jonker et al., 2014). In contrast to the current findings, this large, longitudinal study of 1084 adolescents observed that CRP was not associated with memory or executive function at a two-year follow-up. There are several possible explanations for why our findings do not concur with this study. Firstly, our analyses were cross-sectional; it is possible that within adolescent samples (where cognitive function might be expected to change over time) CRP might be associated with *concurrent* cognitive performance only. Secondly, the study by Jonker and colleagues included only a single measure of executive functioning (the self-ordered pointing task, (Ross et al., 2007)), which may be less sensitive to the effects of inflammation. Finally, our sample was enriched for children presenting with psychopathology; we might therefore expect some individuals within our sample to be characterised by poorer cognitive performance relative to the general population. However, with the exception of scores on the WRAML2 visual memory index, mean scores on all cognitive subtests were higher than the manual-reported means based on normative data (see Table 1). Moreover, as noted above, we found no evidence to suggest that psychopathology moderated the relationship between CRP and cognitive performance.

### 4.1 Potential mechanisms

Our findings provide tentative support for the suggestion that peripheral inflammation, as estimated via salivary CRP, might contribute to impairments in cognitive function. However, the mechanisms underlying this process have not been elucidated fully. Whilst it was previously thought that the brain was protected from the effects of systemic inflammation and immune responses, this has been revised in light of recent evidence that the brain responds to peripheral inflammation and that CRP has neurotoxic properties (for a review see (Warnberg et al., 2009)). In the current study, we observed that salivary CRP was specifically associated with performance on the letter fluency task and the colour-word interference test (similar to the Stroop test; (Stroop, 1935)). Meta-analyses of functional MRI studies indicate that these tests are related to particular brain regions, largely those located in the frontal lobe. Specifically, letter fluency performance is associated with activation of the left inferior/middle frontal gyrus, bilateral anterior cingulate, and the right insula/frontal lobe (Wagner et al., 2014), whilst the anterior cingulate and inferior frontal junction (bridging the inferior frontal sulcus and the inferior precentral sulcus) as well as other frontal regions have been implicated in the colour-word Stroop test (Cieslik et al., 2015, Derrfuss et al., 2005). Longitudinal MRI studies indicate that the brain continues to grow and reorganise throughout adolescence and that the frontal brain regions are among the last to fully mature (Johnson et al., 2009). Thus, one potential reason for why we observed significant associations between salivary CRP and these specific executive functioning measures in this adolescent sample is that the frontal brain regions that subserve these cognitive abilities are under-developed and therefore particularly vulnerable to the effects of CRP. However, it is important to note that in this cross-sectional study it is not possible to determine that CRP has a causal effect on cognitive impairment. Moreover, in the absence of blood-derived CRP data, our findings cannot provide direct evidence of a relationship between systemic inflammation and cognition among relatively healthy adolescents; thus, the mechanisms we propose here should be treated as speculative.

Given that none of the participants exhibited high CRP values indicative of acute infection (see Section 2.2.3), one important question relates to how the association between ‘moderately elevated’ salivary CRP and executive function should be interpreted. Our finding that salivary CRP was not related to recent health problems is consistent with previous studies of healthy adults (Ouellet-Morin et al., 2011, Out et al., 2012) and implies that the association is not driven by poor general health. Accumulated evidence indicates that elevated CRP in later life is predicted by a range of prenatal exposures [e.g., maternal depression and social adversity (Plant et al., 2016, Slopen et al., 2015)], and childhood adversities [e.g., trauma/maltreatment, bullying, and economic/social disadvantage (Baumeister et al., 2016, Copeland et al., 2014, Slopen et al., 2015)]. Thus, the associations that we observed in this sample of children enriched for psychopathology might reflect a general effect of childhood adversity on both inflammation and cognitive functioning. Further research, utilising larger samples, is needed to explore the potentially mediating role of childhood adversity.

### 4.2 Implications

Our findings lend further credence to the notion that the relationships between CRP and cognitive impairments observed among individuals with psychiatric disorder, older adults, and individuals with underlying physical health problems are not merely epiphenomena. Whilst the associations observed in these populations may be confounded by illness chronicity, medication effects, or neuroanatomical changes, such confounders are minimised in this sample of adolescents enriched for psychopathology. Moreover, our findings show that these associations are present in early life before the onset of severe psychiatric disorders. Collectively, our findings and those previous studies, tentatively suggest that novel treatments targeting systemic inflammation might lead to a reduction in persistent cognitive symptoms among psychiatric patients. Such treatment developments are welcomed given that the cognitive features that characterise psychiatric disorders often show only small-to-moderate responses to pharmacological treatments (Mishara and Goldberg, 2004, Rosenblat et al., 2015). One such candidate might be statin therapy, administration of which has been found to attenuate circulating CRP concentration (Albert et al., 2001) and has also been associated with a reduction in cognitive symptoms among individuals with Alzheimer’s disease (Sparks et al., 2006). It remains a question for future research whether statin therapy might induce significant improvement in cognitive symptoms among those already diagnosed with psychiatric disorder, possibly by reducing circulating CRP concentration.

### 4.3 Limitations

Several limitations must be noted. Firstly, the current study is limited by the relatively small sample size, which may have reduced our ability to detect significant associations. Secondly, in this small sample, ‘psychopathology’ was defined as a score in the borderline/clinical range of the YSR and at least one somewhat/certainly true psychotic-like experience. Thus, our ability to detect significant interaction effects between CRP and psychopathology may have been limited by the fact that we used a leniently-defined threshold for psychopathology. A third limitation relates to the fact that we examined only CRP levels in saliva. A previous study using blood-derived markers observed that poor cognitive performance was associated with elevated inflammatory cytokines, but not CRP, among patients with depression (Goldsmith et al., 2016). Our failure to find significant associations between elevated CRP and memory performance, or any moderating effect of psychopathology, might be because CRP (either in saliva or blood) is not a sufficiently sensitive measure of inflammation. Finally, whilst saliva samples were typically obtained within one week of cognitive assessments, this was not always possible (mean lapse of time 1.4 months). As CRP levels may change over time, this delay may have reduced our ability to find significant associations between CRP and cognitive function.

### 4.4 Conclusions

Consistent with studies examining serum/plasma CRP among patients with psychiatric disorders, older adults, and individuals with physical health conditions, we have demonstrated that elevated salivary CRP is associated with poorer performance on measures of executive function in this sample of children aged 11–14 years that is enriched for those presenting with psychopathology. In contrast to expectations, psychopathology did not appear to modify the effect of CRP on cognition. Our findings obtained in this sample of relatively healthy adolescents, where confounding factors such as medication use, illness chronicity, and neuroanatomical changes are minimised, provide further evidence to suggest that the association between CRP and cognitive function is not merely an epiphenomenon, though the absence of blood-derived CRP data prevents us from drawing definitive conclusions regarding the role of peripheral inflammation in cognitive function. Nevertheless, the robust associations that we observed in this sample of adolescents between salivary CRP and executive function, which withstood adjustment for a range of factors, provide exciting prospects for intervention strategies aimed at reducing cognitive impairments in vulnerable populations.
