# Supplementary material for: The relationship between salivary C-reactive protein and cognitive function in children aged 11–14 years: Does psychopathology have a moderating effect?
Source: Brain Behav Immun. 2017 Nov;66:221–9. doi: 10.1016/j.bbi.2017.07.002 (PMC5773474; doi:10.1016/j.bbi.2017.07.002)
Supplement: Supplementary data 1 [file mmc1.docx]

# Supplementary Material

## Supplementary Analyses 1: Factors associated with salivary CRP

## Sample availability

A total of 659 saliva samples were obtained from 107 participants and assayed for CRP. Of these, 52 (7.9%) were found to have coefficient of variation (CV) values exceeding 20%, indicating substantial variation between duplicate assay values, and were therefore excluded. CRP values ranged from 75.0 to 367,859.4 pg/ml (mean: 3,943.5; SD: 174,27.9). Two samples, which yielded CRP values 47 and 93 times larger than the mean value (186,231.7 and 367,859.4 pg/ml, respectively), were considered outliers and therefore excluded. Data checks performed on the remaining samples (N=605) indicated that these data were not normally distributed. Applying a log transformation improved the distribution, although the kurtosis statistic remained high (6.08). Subsequent regression analyses were therefore performed with robust standard errors.

- 1. *Analyses*
     1. *Effect of methodological factors on salivary CRP using all available samples*

Mixed effects linear regression analyses were performed to examine the effect of all methodological factors (i.e., sample time, sample day, awakening time, length of time in storage), on salivary CRP levels, with participant ID entered as a random effect to account for clustering at the participant level. Results of these analyses are presented in Supplementary Table 1, with descriptive statistics (mean ± SD) provided for raw and log-transformed CRP values. Analyses indicated a significant effect of sampling time; relative to the awakening sample, CRP values at all other time-points were significantly lower (*p*≤0.02). CRP values for all non-awakening time-points did not differ significantly from each other (*p*>0.05, data not shown).

**Supplementary Table 1.** Association of methodological factors with salivary C-reactive protein

|  | No. available samples ^a^ | | Raw CRP value | | Log CRP value | | Regression analyses ^d^ | | |
| --- | --- | --- | --- | --- | --- | --- | --- | --- | --- |
|  | N | (%) | Mean | (SD) | Mean | (SD) | *B* | 95% CI | *p* |
| Sample time |  |  |  |  |  |  |  |  |  |
| Awakening | 91 | (15.0) | 4855.1 | (7673.0) | 7.8 | (1.1) | ------- | (reference) | ------ |
| 15 min post-awakening | 104 | (17.2) | 2867.4 | (5324.4) | 7.5 | (0.8) | -0.35 | **(-0.53 to -** **0.18)** | **<0.001** |
| 30 min post-awakening | 96 | (15.9) | 2731.6 | (3984.7) | 7.6 | (0.7) | -0.24 | **(-0.42 to -0.07)** | **0.007** |
| 60 min post-awakening | 98 | (16.2) | 3033.1 | (7011.8) | 7.6 | (0.7) | -0.24 | **(-0.44 to -0.05)** | **0.02** |
| 12:00 h | 111 | (18.3) | 2591.8 | (4476.2) | 7.5 | (0.7) | -0.30 | **(-0.51 to -0.08)** | **0.006** |
| 20:00 h | 105 | (17.4) | 2403.9 | (2821.8) | 7.5 | (0.6) | -0.30 | **(-0.52 to -0.09)** | **0.005** |
| Sample day |  |  |  |  |  |  |  |  |  |
| Day 1 | 424 | (70.1) | 2726.4 | (3661.9) | 7.6 | (0.7) | ------- | (reference) | ------ |
| Day 2 | 181 | (29.9) | 3776.8 | (8196.5) | 7.6 | (0.9) | **-0.40** | **(-0.60 to -0.20)** | **<0.001** |
| Time of awakening ^b^ |  |  |  |  |  |  |  |  |  |
| Before 08:00 AM | 242 | (42.8) | 3451.4 | (7466.9) | 7.6 | (0.9) | ------- | (reference) | ------ |
| 08:00 – 09:00 AM | 139 | (24.6) | 2489.2 | (2512.6) | 7.6 | (0.6) | -0.02 | (-0.20 to 0.16) | 0.79 |
| After 09:00 AM | 185 | (32.7) | 3095.6 | (4275.6) | 7.6 | (0.8) | -0.02 | (-0.33 to 0.30) | 0.90 |
| Length of time in storage ^c^ |  |  |  |  |  |  |  |  |  |
| < 3 years | 169 | (36.9) | 2604.6 | (3464.0) | 7.5 | (0.7) | ------- | (reference) | ------ |
| 3 – 4 years | 163 | (35.6) | 2963.5 | (3212.5) | 7.7 | (0.7) | 0.19 | (-0.17 to 0.54) | 0.30 |
| > 4 years | 126 | (27.5) | 2205.0 | (2155.1) | 7.4 | (0.7) | -0.06 | (-0.45 to 0.33) | 0.75 |

Note. SD, standard deviation; *B:* unstandardized regression coefficient from mixed effects model. ^a^ Total number of saliva samples included in analyses (N=605). ^b^ Total number of time of awakening observations (n=566). ^c^ Total number length of time in storage observations (n=458). ^d^ Multivariable model including all methodological factors (i.e., sample time, sample day, awakening time, lapse of time between sample collection and assay). All analyses performed with robust standard errors using log transformed CRP.

CRP values for samples collected on Day 2 were significantly lower than those obtained on Day 1 (*B =* -0.40, *p<*0.001); however, neither awakening time nor length of time in storage were associated with CRP (*p*>0.05 for both). Post-hoc analyses indicated that the awakening sample was significantly higher than all other time-points on Day 1 but not on Day 2 (see Figure 1). The different patterns observed across Day 1 and Day 2 were not explained by a difference in waking times, which did not differ across the two sampling days (*t*=0.72, *p=*0.47).

**Supplementary Fig 1.** Diurnal profile of salivary C-reactive protein (CRP) across two testing days

- - 1. *Correlations between salivary CRP values at individual time-points*

Non-parametric correlation analyses were performed to examine relationships between salivary CRP levels at multiple time-points (Supplementary Table 2). Moderate-to-high correlations were observed across all Day 1 samples (*p*<0.05). A similar pattern was observed for Day 2 samples, with the exception that 12:00 h samples were moderately, but not significantly, correlated with samples obtained at 30 min or 60 min post-awakening or 20:00 h.

**Supplementary Table 2.** Correlations between salivary CRP values within and across sampling days

|  |  |  | Day 1 | | | | | | Day 2 | | | | | |
| --- | --- | --- | --- | --- | --- | --- | --- | --- | --- | --- | --- | --- | --- | --- |
|  | Time |  | Awake | 15 min | 30 min | 60 min | 12:00 h | 20:00 h | Awake | 15 min | 30 min | 60 min | 12:00 h | 20:00 h |
| Day 1 | Awake | *rho* | 1.000 |  |  |  |  |  |  |  |  |  |  |  |
|  |  | *N* | 62 |  |  |  |  |  |  |  |  |  |  |  |
|  | 15 min | *rho* | 0.782* | 1.000 |  |  |  |  |  |  |  |  |  |  |
|  |  | *N* | 59 | 72 |  |  |  |  |  |  |  |  |  |  |
|  | 30 min | *rho* | 0.773* | 0.861* | 1.000 |  |  |  |  |  |  |  |  |  |
|  |  | *N* | 57 | 63 | 66 |  |  |  |  |  |  |  |  |  |
|  | 60 min | *rho* | 0.693* | 0.853* | 0.786* | 1.000 |  |  |  |  |  |  |  |  |
|  |  | *N* | 55 | 64 | 60 | 70 |  |  |  |  |  |  |  |  |
|  | 12:00 h | *rho* | 0.610* | 0.512* | 0.591* | 0.504* | 1.000 |  |  |  |  |  |  |  |
|  |  | *N* | 57 | 66 | 63 | 65 | 83 |  |  |  |  |  |  |  |
|  | 20:00 h | *rho* | 0.659* | 0.799* | 0.749* | 0.735* | 0.483* | 1.000 |  |  |  |  |  |  |
|  |  | *N* | 56 | 63 | 59 | 65 | 66 | 71 |  |  |  |  |  |  |
| Day 2 | Awake | *rho* | 0.429 | 0.667* | 0.857* | 0.450 | 0.074 | 0.714* | 1.000 |  |  |  |  |  |
|  |  | *N* | 7 | 9 | 7 | 9 | 17 | 8 | 31 |  |  |  |  |  |
|  | 15 min | *rho* | 0.429 | 0.617 | 0.286 | 0.783* | 0.123 | 0.867* | 0.671* | 1.000 |  |  |  |  |
|  |  | *N* | 7 | 9 | 7 | 9 | 17 | 9 | 28 | 32 |  |  |  |  |
|  | 30 min | *rho* | 0.143 | 0.524 | 0.486 | 0.667 | 0.065 | 0.600 | 0.614* | 0.787* | 1.000 |  |  |  |
|  |  | *N* | 7 | 8 | 6 | 8 | 16 | 9 | 26 | 28 | 30 |  |  |  |
|  | 60 min | *rho* | -0.029 | 0.314 | 0.500 | 0.333 | 0.242 | 0.714 | 0.626* | 0.695* | 0.851* | 1.000 |  |  |
|  |  | *N* | 6 | 6 | 5 | 8 | 13 | 7 | 24 | 24 | 24 | 28 |  |  |
|  | 12:00 h | *rho* | 0.429 | 0.600 | -0.800 | 0.314 | 0.376 | 0.607 | 0.494* | 0.609* | 0.354 | 0.338 | 1.000 |  |
|  |  | *N* | 6 | 6 | 5 | 6 | 14 | 7 | 23 | 22 | 23 | 20 | 28 |  |
|  | 20:00 h | *rho* | -0.393 | -0.095 | 0.179 | 0.067 | 0.191 | 0.300 | 0.412* | 0.409* | 0.455* | 0.444* | 0.372 | 1.000 |
|  |  | *N* | 7 | 8 | 7 | 9 | 17 | 9 | 28 | 29 | 29 | 26 | 26 | 34 |

Note. rho: Spearman’s rank correlation coefficient; N: number of observations; * *p*<0.05.

Correlations between salivary CRP levels obtained across testing days are also shown in Supplementary Table 2. For all time-points, salivary CRP samples obtained on Day 1 were not associated significantly with those obtained at the same time-point on Day 2 (*p*>0.05). Correlational analyses therefore support the results of the mixed effects model by demonstrating that, for any given time-point, salivary CRP samples are not stable across testing days. However, it is important to note the small N available for each analysis.

## Effect of participant factors on salivary CRP using all available samples

Mixed effect linear regression analyses with participant ID included as a random effect were next performed on the total number of available samples to examine the effect of each participant demographic factor on salivary CRP. All analyses were adjusting for time and day of sampling (Supplementary Table 3). Results indicated that salivary CRP was not significantly associated with participant age, sex, BMI, ethnicity, socioeconomic status, a family history of psychosis, or recent health problems. In contrast, participants who reported recent dental problems had higher salivary CRP levels than those who did not (*B*=0.46, *p*=0.03).

**Supplementary Table 3.** Effect of participant factors on salivary C-reactive protein

|  | No. available samples | | Raw CRP value | | Log CRP value | | Regression analyses ^e^ | | |
| --- | --- | --- | --- | --- | --- | --- | --- | --- | --- |
|  | N | (%) | Mean | (SD) | Mean | (SD) | *B* | 95% CI | *p* |
| Age |  |  |  |  |  |  |  |  |  |
| <13 years | 257 | (42.6) | 3306.5 | (5764.9) | 7.6 | (0.9) | ------- | (reference) | ------ |
| 13-14 years | 207 | (34.3) | 2428.3 | (3025.8) | 7.5 | (0.7) | -0.17 | (-0.45 to 0.11) | 0.23 |
| >14 years | 140 | (23.2) | 3468.5 | (7290.3) | 7.6 | (0.8) | 0.03 | (-0.34 to 0.40) | 0.85 |
| Sex |  |  |  |  |  |  |  |  |  |
| Male | 277 | (45.8) | 3766.4 | (7372.7) | 7.7 | (0.9) | ------- | (reference) | ------ |
| Female | 328 | (54.2) | 2427.8 | (2837.8) | 7.5 | (0.7) | -0.20 | (0.45 to 0.06) | 0.13 |
| BMI ^b^ |  |  |  |  |  |  |  |  |  |
| Healthy weight | 483 | (81.9) | 3078.7 | (5733.5) | 7.6 | (0.8) | ------- | (reference) | ------ |
| Overweight | 85 | (14.4) | 2689.7 | (3868.8) | 7.5 | (0.7) | -0.04 | (-0.39 to 0.32) | 0.85 |
| Obese | 22 | (3.7) | 3303.3 | (3808.3) | 7.7 | (0.9) | 0.06 | (-0.52 to 0.65) | 0.83 |
| Ethnicity |  |  |  |  |  |  |  |  |  |
| White British | 205 | (33.9) | 2818.5 | (5923.5) | 7.5 | (0.8) | ------- | (reference) | ------ |
| White other | 154 | (25.4) | 2206.9 | (1721.6) | 7.5 | (0.6) | -0.06 | (-0.36 to 0.24) | 0.70 |
| Black African or Caribbean | 66 | (10.9) | 1891.0 | (1117.2) | 7.4 | (0.5) | -0.06 | (-0.38 to 0.25) | 0.69 |
| Other | 180 | (29.8) | 4428.5 | (7346.7) | 7.8 | (1.0) | 0.28 | (-0.08 to 0.64) | 0.13 |
| Socioeconomic status |  |  |  |  |  |  |  |  |  |
| Higher managerial, administrative and professional | 394 | (65.1) | 2556.7 | (4503.0) | 7.5 | (0.7) | ------- | (reference) | ------ |
| Intermediate | 140 | (23.1) | 4252.8 | (6760.3) | 7.7 | (1.0) | 0.26 | (-0.08 to 0.61) | 0.14 |
| Routine and manual | 71 | (11.7) | 3336.3 | (6844.5) | 7.6 | (0.8) | 0.18 | (-0.12 to 0.48) | 0.24 |
| Any recent health problem ^c^ |  |  |  |  |  |  |  |  |  |
| No | 330 | (57.6) | 2697.0 | (3941.8) | 7.5 | (0.7) | ------- | (reference) | ------ |
| Yes | 243 | (42.4) | 3648.5 | (7211.3) | 7.6 | (0.9) | 0.11 | (-0.17 to 0.39) | 0.44 |

Supplementary Table 3, continued

|  | No. available samples | | Raw CRP value | | Log CRP value | | Regression analyses ^e^ | | |
| --- | --- | --- | --- | --- | --- | --- | --- | --- | --- |
|  | N | (%) | Mean | (SD) | Mean | (SD) | *B* | 95% CI | *p* |
| Any recent dental problem ^d^ |  |  |  |  |  |  |  |  |  |
| No | 479 | (83.6) | 2854.6 | (5592.0) | 7.5 | (0.8) | ------- | (reference) | ------ |
| Yes | 94 | (16.4) | 4353.9 | (5387.4) | 7.9 | (0.9) | **0.46** | **(0.06 to 0.86)** | **0.03** |
| Family history of psychosis |  |  |  |  |  |  |  |  |  |
| No | 457 | (75.5) | 3111.7 | (5798.2) | 7.6 | (0.8) | ------- | (reference) | ------ |
| Yes | 148 | (24.5) | 2821.4 | (4177.5) | 7.5 | (0.8) | -0.07 | (-0.37 to 0.22) | 0.63 |

Note. SD, standard deviation; BMI: Body Mass Index; *B:* unstandardized regression coefficient from mixed effects model. ^a^ Total number of saliva samples included in analyses (N=605). ^b^ Total number of BMI observations (n=590). ^c^ Total number of recent health problem observations (n=573). ^d^ Total number of recent dental observations (n=573). ^e^ Effect of each participant factor adjusted for methodological factors significantly associated with CRP (i.e., sample time and sample day). All analyses performed with robust standard errors using log transformed CRP.

## Supplementary Analyses 2: Association of psychopathology and salivary CRP

Linear regression analyses were performed to examine the effect of each psychopathology variable on salivary CRP (Supplementary Table 4). Adjusting for sampling day and dental problems, neither internalising symptoms, externalising symptoms, nor psychotic-like experiences were significantly associated with salivary CRP values (*p*>0.05). The pattern of results was unchanged after including all psychopathology variables in a single model.

**Supplementary Table 4.** Associations between psychopathology and salivary CRP among children aged 11-14 years

|  | Descriptive statistics for salivary CRP among those with and without psychopathology ^a^ | | | | Effect psychopathology on salivary CRP adjusted for methodological and participant factors ^b^ | | | | | Effect of psychopathology on salivary CRP adjusted for methodological and participant factors and other psychopathology ^c^ | | | |
| --- | --- | --- | --- | --- | --- | --- | --- | --- | --- | --- | --- | --- | --- |
|  | Present  Mean (SD) | | Absent  Mean (SD) | | *β* | *B* | (95% CI) | *p* | *β* | | *B* | (95% CI) | *p* |
| YSR Internalising symptoms | 44.6 | (9.2) | 43.2 | (11.7) | 0.05 | 1.44 | (-3.43 to 6.31) | 0.56 | 0.04 | | 1.29 | (-4.68 to 7.26) | 0.67 |
| YSR Externalising symptoms | 46.0 | (13.6) | 43.2 | (11.0) | 0.07 | 2.59 | (-5.19 to 10.37) | 0.51 | 0.06 | | 2.21 | (-6.27 to 10.70) | 0.61 |
| Psychotic-like experiences | 43.4 | (11.8) | 43.7 | (11.0) | -0.03 | -0.62 | (-5.07 to 3.82) | 0.78 | -0.03 | | -0.77 | (-5.85 to 4.31) | 0.76 |

Note. YSR: Youth Self-Report; SD: standard deviation; *β* standardised beta coefficient from linear regression analysis; *B*: unstandardised regression coefficients; CI: confidence interval. All analyses performed with robust standard errors. ^a^ Square root transformed CRP variable; ^b^ Adjusted for day of saliva sample collection (Day 1 vs. Day 2) and recent dental problems; ^c^ additionally adjusted for all other psychopathology variables.
